# From Carcinogenesis to Drug Resistance: The Multifaceted Role of Oxidative Stress in Head and Neck Cancer

**DOI:** 10.3390/cancers17203295

**Published:** 2025-10-11

**Authors:** Enas Bani-Ahmad, Joshua Dass, Crispin R. Dass

**Affiliations:** 1 Curtin Medical School, Curtin University, Kent St, Bentley 6102, Australia; e.bani-ahmad@postgrad.curtin.edu.au (E.B.-A.); joshua.dass@health.wa.gov.au (J.D.); 2 Curtin Medical Research Institute, Curtin University, Kent St, Bentley 6102, Australia; 3 Faculty of Pharmacy, Jordan University of Science and Technology, Irbid 3030, Jordan; 4 Department of Radiation Oncology, Sir Charles Gairdner Hospital, Nedlands 6009, Australia; 5 Faculty of Pharmacy, Silpakorn University, Nakhon Pathom 73000, Thailand

**Keywords:** head and neck squamous cell carcinoma, oxidative stress, reactive oxygen species, antioxidant systems

## Abstract

**Simple Summary:**

Head and neck cancer is one of the most common cancers worldwide and is often linked to tobacco use, alcohol consumption, and certain viral infections. These factors increase harmful molecules called reactive oxygen species, which can damage cells but can also be used to destroy cancer cells. This review explores the dual role of these molecules in cancer—helping tumors grow in some cases, while also offering a way to eliminate cancer cells through treatment. We describe how cancer cells protect themselves from this damage by activating defense systems that can make them resistant to therapy. Understanding these mechanisms may guide the development of more effective future treatments that precisely target cancer cells, minimize side effects, and improve patient survival and quality of life.

**Abstract:**

**Objectives**: This review examines the role of oxidative stress in the survival, apoptosis, and therapy resistance of head and neck squamous cell carcinoma (HNSCC) cells, with a focus on how redox imbalance influences tumour progression and treatment outcomes. **Methods**: A literature search was conducted in Scopus using the keywords head and neck squamous cell carcinoma, oxidative stress, reactive oxygen species (ROS), and antioxidant systems. Articles published in English were included, without restrictions on publication year. Reviews, clinical studies, and experimental research addressing oxidative stress mechanisms in HNSCC were considered, while non-English papers and studies unrelated to HNSCC were excluded. **Key Findings**: ROS exhibit dual effects in HNSCC, promoting tumour growth and DNA damage while also inducing apoptosis through molecular interactions. Elevated ROS contribute to drug resistance by inhibiting apoptosis, altering autophagy, and enhancing proliferation. Cancer cells counteract this via adaptive antioxidant responses involving transcriptional regulation and upregulation of enzymatic defences. Major risk factors for HNSCC—alcohol, tobacco, and high-risk HPV infection—disrupt redox homeostasis, underscoring the central role of oxidative stress in both carcinogenesis and therapy response. **Conclusions**: Oxidative stress plays a context-dependent role in HNSCC progression and treatment resistance. Targeting redox-regulatory pathways may provide therapeutic benefit. This review synthesizes recent insights on ROS-mediated mechanisms, highlighting potential strategies for improving HNSCC management beyond existing literature.

## 1. Oxidative Stress and Cancer

Cell death is essential for maintaining tissue homeostasis, but when dysregulated, it can contribute to pathological processes such as cancer development [1]. Regulated cell death (RCD) happens via specific molecular pathways, while accidental cell death (ACD) occurs randomly without regulation [2]. One of the major triggers of RCD is oxidative stress, which results from an imbalance between oxidant production and antioxidant defenses, leading to the accumulation of reactive oxygen species (ROS) [1].

ROS are highly reactive molecules generated mainly by mitochondria and NADPH oxidases [3]. However, they may also arise from many internal organelles and external stressors, such as inflammatory cells, radiation, chemotherapy, tobacco, and alcohol. Table 1 delineates the principal intracellular and extracellular origins of ROS pertinent to cancer and head and neck squamous cell carcinoma (HNSCC). Among ROS-producing enzymes, several appear particularly relevant in HNSCC. 15-LOX-2 expression is significantly reduced, and this reduction may promote proliferation in head and neck carcinoma, suggesting that 15-LOX-2 could serve as a potential biomarker in HNSCC [4]. In contrast, NOX enzymes are frequently overexpressed in HNSCC, where they enhance ROS generation and contribute to tumour progression and therapy resistance [5]. Furthermore, xanthine oxidase (XO) activity is elevated in HNSCC, leading to increased oxidative stress and highlighting its potential as a therapeutic target [6,7].

In cancer, ROS have paradoxical effects. Moderate ROS levels can promote tumourigenesis by inducing DNA damage, genomic instability, and proliferative signaling [3,8,9]. Conversely, when ROS levels exceed the cell’s antioxidant capacity, they can overwhelm redox defenses and induce cancer cell death, while often leaving normal cells relatively unharmed [10,11]. This “double-edged sword” behavior highlights ROS as potential therapeutic targets: while persistent low-to-moderate ROS may drive malignancy, pharmacologically or therapeutically induced ROS can promote tumour suppression.

**Table 1:** Sources of Reactive Oxygen Species (ROS).

Source	Mechanism	Pathway to Increase ROS Production	Type	Ref
Mitochondrial Dysfunction	The electron transport chain is responsible for massive production of O_2_^• −^	Complex I/III dysfunction. Enzymes glycerol-3-phosphate dehydrogenase, 2-oxoglutarate, pyruvate dehydrogenase, and ETFQOR.	Intracellular sources of ROS	[12,13,14,15]
NADPH oxidases	Enzymatic ROS production	NOX1, NOX2, and NOX4 overexpression.	Intracellular sources of ROS	[12,16]
Xanthine Oxidase	Catalyses the oxidative hydroxylation of hypoxanthine to xanthine and xanthine to uric acid. O_2_ quickly accepts electrons derived from hypoxanthine and xanthine oxidation by XO to generate O_2_^• −^ and H_2_O_2_	XO is upregulated in aging, which is associated with oxidative stress, immunosenescence, and inflammation	Intracellular sources of ROS	[17,18,19]
Uncoupled NO Synthase	NO can be generated by three different isoforms of the enzyme NO synthase (NOS). The isozymes are referred to as neuronal nNOS (or NOS I), inducible form iNOS (or NOS II), and endothelial eNOS (or NOS III)	Uncoupled eNOS produces superoxide, which scavenges NO to form ONOO¯, a short-lived and powerful oxidant promoting oxidation and nitration reactions that affect different biomolecules, including lipids, proteins, and DNA.	Intracellular sources of ROS	[20,21]
Endoplasmic Reticulum	ERO1	ER dysfunction	Intracellular sources of ROS	[22,23]
Cytochrome p450	Four components form the CYP catalytic system: the substrate, a P450 enzyme (an enzyme that performs oxidative catalysis), a redox agent (performs electron transfer—NADPH cytochrome P450 reductase, and cytochrome b5), and the NADPH cofactor (provides reducing equivalents)	The aging process is associated with a decline in liver function, leading to changes in biotransformation	Intracellular sources of ROS	[24,25]
Peroxidases	Lactoperoxidase, myeloperoxidase and eosinophil peroxidase. These enzymes can oxidise iodide, bromide, and chloride ions to form reactive halogen species such as HOCl	Contributes to oxidative stress when it is persistent and eventually leads to protein, lipid, and DNA damage.	Intracellular sources of ROS	[26,27]
Cyclooxygenases	The enzyme cyclooxygenase is responsible for the metabolism of arachidonic acid by generating prostaglandin precursors, which contribute to inflammation, ROS production, and lipid oxygenation	COX-2 upregulation	Intracellular sources of ROS	[28,29]
Lipid Oxidases	The ROS formation process occurs when Fe^3+^ present in activated LOX is reduced by the contact of this enzyme with its substrate	The aging process is associated with an increase in LOX expression and activity, thus generating a significant increase in ROS.	Intracellular sources of ROS	[30,31]
Air pollution	Contains numerous toxic agents such as metals and other chemicals such ROS/RNS that leading to local or systemic oxidative stress and inflammation	Pollution introduces harmful particles that cause oxidative stress, first using up antioxidant defenses in lung cells. The body adapts by activating protective genes (like Nrf2). High exposure: If stress continues and overwhelms defenses, it triggers inflammation (via NF-κB) and further ROS production, leading to cell damage or death.	Exogenous sources of ROS	[32]
Chemotherapeutic and radiation	Enhancing oxidative damage in proteins, lipids, and DNA.	Increased lipid peroxidation and decreased antioxidant levels, including tissue GSH	Exogenous sources of ROS	[33,34]

Electron transfer flavoprotein Q oxidoreductase: ETFQOR, NADPH oxidase: NOX, Xanthine Oxidase: XO, ER oxirreductin-1: ERO1, Endoplasmic Reticulum: ER, cyclooxygenase: COX, Lipid Oxidases: LOX.

Building on this principle, conventional anticancer treatments such as radiotherapy and chemotherapy rely on ROS to induce cytotoxicity in HNSCC. Ionising radiation generates free radicals that damage DNA, proteins, and lipids, ultimately triggering apoptosis [35]. Similarly, Chemotherapeutic drugs, such as platinum compounds, elevate mitochondrial ROS generation or impair antioxidant defence systems [36,37]. Despite their effectiveness, a continual difficulty is attaining selective toxicity-eliminating HNSCC cells while preserving adjacent healthy tissues.

This review focuses on the dual role of ROS in HNSCC, a malignancy closely associated with oxidative stress resulting from risk factors including tobacco, alcohol, and human papillomavirus (HPV) infection, describing how reactive oxygen species (ROS) can either promote tumour growth or suppress it, depending on the context. We also discuss how chemotherapy and radiotherapy exploit ROS to kill cancer cells and summarise clinical evidence on ROS-targeted therapies, aiming to bridge laboratory research with potential applications in patient care.

## 2. HNSCC and Oxidative Stress

HNSCC ranks as the sixth most common cancer globally, with 890,000 new cases and 450,000 deaths reported in 2018. Its incidence is continuing to rise and is expected to increase by around 30%, reaching approximately 1.08 million new cases per year by 2030 [38]. Despite improvements in surgical, chemotherapy, and radiotherapy approaches, the five-year survival rate remains low due to high rates of recurrence and treatment resistance [39]. Key risk factors—such as tobacco use, alcohol consumption, and infection with high-risk human papillomavirus (HPV) contribute to cancer development in part by triggering oxidative stress, highlighting the need to better understand redox regulation in HNSCC. To date, relatively few studies have directly examined the relationship between oxidative stress and HNSCC [40,41]. Nevertheless, evidence indicates that elevated oxidative stress, coupled with reduced antioxidant defenses, is associated with higher HNSCC incidence and more aggressive disease, partly due to oxidative DNA damage [40].

The role of ROS in HNSCC is paradoxical. Moderate ROS levels can promote neoplastic transformation and DNA damage, supporting tumour survival and proliferation, whereas excessive ROS can trigger apoptosis through interactions with key signaling molecules [10,42]. Therapeutic strategies in HNSCC therefore exploit both aspects: some aim to reduce oxidative stress to limit tumour-promoting effects, while others intentionally elevate ROS beyond a critical threshold to selectively induce cancer cell death.

Although cancer cells often adapt to harsh environments and develop resistance to chemotherapy, their reliance on tightly regulated ROS levels represents a potential vulnerability. Each cell has a tolerance threshold: ROS levels below it promotes survival, while levels above it induces death. ROS-modulating agents under investigation seek to push ROS beyond this threshold in HNSCC cells, bypassing defense mechanisms and reducing both drug resistance and side effects [43]. For this reason, ROS remains an important and promising therapeutic target in HNSCC.

## 3. Oxidative Stress and Drug Resistance in HNSCC

HNSCC exhibits significant heterogeneity based on HPV status, which influences treatment response and resistance. HPV-positive HNSCC generally harbors fewer genetic mutations and is associated with a more favorable prognosis, often showing increased sensitivity to radiation and immunotherapy [44]. In contrast, HPV-negative HNSCC typically has a higher mutational burden and complex molecular alterations, contributing to intrinsic resistance to standard treatments and poorer clinical outcomes [45]. Consequently, HPV-negative tumours are more likely to develop multidrug resistance (MDR), while HPV-positive tumours tend to be more treatment-sensitive [46,47]. Beyond the type of resistance, understanding the underlying molecular mechanisms is critical for developing effective therapies.

ROS play a central role in mediating drug resistance by neutralising anticancer agents, inhibiting apoptosis, modulating autophagy, and promoting proliferation [43]. Several ROS-sensitive signaling pathways contribute to this effect. The PI3K/AKT/mTOR pathway is crucial in HNSCC development and progression, with its activity being ROS-dependent: moderate ROS levels can promote survival, while higher levels may induce apoptosis [48,49]. PTEN, a negative regulator of PI3K/AKT/mTOR, both influences and is influenced by ROS; loss or suppression of PTEN enhances resistance to therapy [50,51]. Similarly, the MEK/ERK/JNK pathways, when activated by ROS, promote invasion and resistance in oral cancers [52,53]. FOXM1 functions as a central transcription factor linking ROS to drug resistance in HNSCC and other head and neck cancers [54,55].

**Figure 1:**
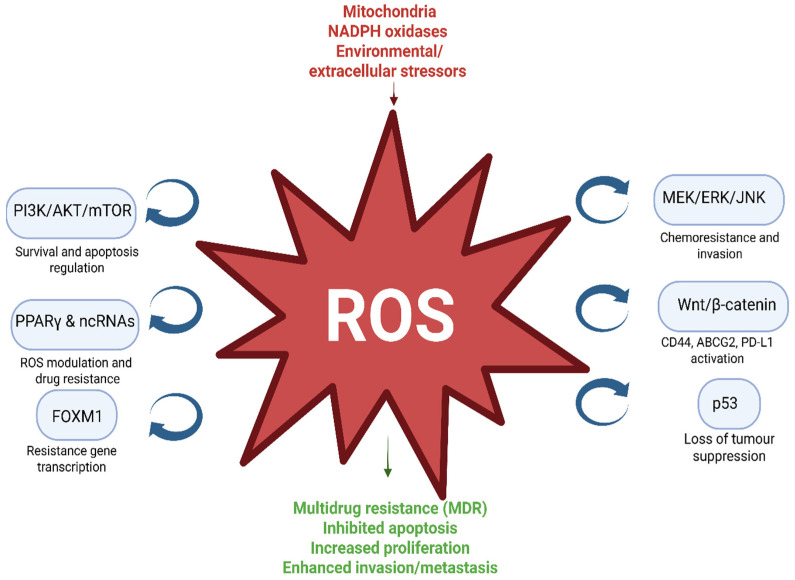
ROS-Driven Signaling Pathways in HNSCC.

Hypoxia-inducible factor (HIF) is another key player: it mediates hypoxia-induced resistance to both conventional and novel anticancer agents [10,56], and elevated HIF levels correlate with poor chemotherapy response in HNSCC [57,58]. Notably, HIF activity is both regulated by ROS and capable of influencing ROS production, creating a feedback loop that supports tumour survival and therapy resistance [59,60]. p53 is a key tumour suppressor that is often mutated in various cancers, including HNSCC [61]. Mutant or dysfunctional p53 enhances ROS accumulation, mTOR pathway activation [61,62], and multidrug resistance (MDR) [63]. Furthermore, Wnt/β-catenin activated by ROS and hypoxia promotes resistance via targets like CD44, ABCG2, and PD-L1 in HNSCC [64]. Other modulators, such as PPARγ and non-coding RNAs, also influence ROS homeostasis and chemoresistance in HNSCC [65,66,67]. Figure 1

Collectively, these pathways create complex feedback loops in which ROS are regulated by key signaling molecules. This dual role of ROS underpins its contribution to tumour survival and therapy resistance, emphasising the importance of understanding disruptions in redox balance for designing strategies to overcome chemoresistance and improve therapeutic outcomes in HNSCC.

ROS are produced from multiple sources (e.g., mitochondria, NOX enzymes, hypoxia, and environmental factors like smoking, alcohol, and therapy). Once generated, ROS activate or inhibit several ROS-sensitive signalling pathways, including PI3K/AKT/mTOR, MEK/ERK/JNK, FOXM1, p53, Wnt/β-catenin, and PPARγ/ncRNAs. These pathways, in turn, regulate key cellular processes such as apoptosis, proliferation, invasion, autophagy, and drug efflux, ultimately leading to multidrug resistance (MDR). Importantly, feedback loops exist in which ROS both regulate and are regulated by these signalling cascades, amplifying resistance mechanisms. Created in BioRender. <Bani-Ahmad>, <Enas>. (2025) https://BioRender.com/12vnv0r (accessed on 28 September 2025).

## 4. Oxidative and Antioxidative Stress in HNSCC

**Figure 2:**
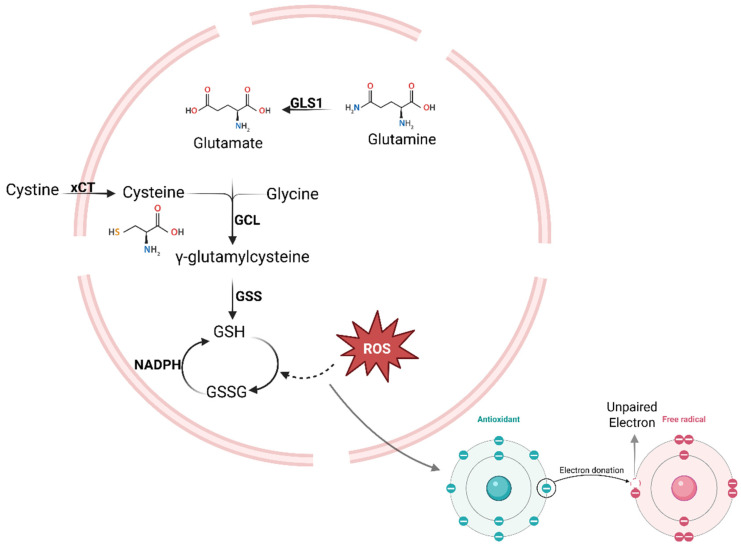
Glutathione (GSH) synthesis and antioxidant function.

Cancer cells, including those in HNSCC, strive to maintain a balance between oxidative and antioxidative stress through adaptive mechanisms involving transcriptional pathways and the upregulation of gene-encoded antioxidant enzymes [68]. ROS levels are tightly regulated by both enzymatic and non-enzymatic antioxidant systems. Key enzymatic antioxidants include superoxide dismutase (SOD), catalase (CAT), glutathione peroxidase (GPx), NADPH-quinone oxidoreductase-1 (NQO1), heme oxygenase-1 (HO-1), thioredoxin (Trx), and sulfiredoxin (Srx), while non-enzymatic antioxidants encompass vitamins C and E, β-carotene, uric acid, and glutathione (GSH) [69,70]. Table 2 summarises these major antioxidant defense mechanisms and highlights differences observed between normal tissues and HNSCC. Among these systems, GSH plays a central role in maintaining redox homeostasis through redox reactions, existing in both reduced (GSH) and oxidised (GSSG) forms. Within cells, GSH can be found in free and protein-bound forms, allowing it to participate flexibly in neutralising ROS and maintaining cellular redox balance. The free form is mostly reduced and is oxidised during oxidative stress to GSSG. Inside cells, GSH neutralises free radicals and becomes GSSG in the process (Figure 2). The GSSG/GSH ratio reflects the cellular redox state: a low ratio indicates effective antioxidative defense, while a high ratio signifies elevated oxidative stress [71].

In HNSCC, variations in GSH and GSSG levels have been observed across tumours. Some studies report lower GSSG/GSH ratios in tumours compared to healthy tissues, suggesting either reduced oxidative stress or enhanced antioxidant defenses [99,100]. Clinically, higher GSSG/GSH ratios have been associated with lymph node involvement (N-positive patients) and increased risk of tumour recurrence, implying that elevated oxidative stress may correlate with more aggressive disease, although definitive links to patient survival remain unclear [68].

Other antioxidant systems can paradoxically support tumour survival and therapy resistance in HNSCC. For instance, Paraoxonase-2 (PON2) is an intracellular antioxidant that regulates mitochondrial ROS and apoptosis. In HNSCC, PON2 is often overexpressed [79], promoting tumour survival by reducing oxidative stress and inhibiting cell death. High PON2 levels are linked to poor prognosis, chemoradiotherapy resistance, and lower overall survival, making it a potential biomarker and therapeutic target, as its inhibition increases ROS-mediated cancer cell death [79,101,102]. Similarly, the NRF2/KEAP1 pathway, a key regulator of antioxidant gene expression, is frequently dysregulated in HNSCC. Persistent NRF2 activation promotes tumour proliferation, metabolic adaptation, and resistance to ROS-induced apoptosis, contributing to cisplatin and radiotherapy resistance in preclinical models [103,104,105,106].

These findings underscore the dual nature of antioxidant mechanisms in HNSCC. While antioxidant systems are essential for maintaining cellular homeostasis and preventing early carcinogenesis, their dysregulation can provide malignant cells with selective advantages, promoting tumour progression and resistance to therapy. The interplay between oxidative and antioxidative stress is therefore complex and context-dependent, representing both a vulnerability and a potential therapeutic target in HNSCC [41].

Cystine is imported into the cell via system xCT and reduced to cysteine. Glutamate and glycine are supplied from glutamine and serine. GSH is synthesised in two ATP-dependent steps and detoxifies reactive oxygen species (ROS) via glutathione peroxidase, forming oxidised glutathione (GSSG), which is regenerated by glutathione reductase using NADPH. GSH also protects proteins and mitochondria from oxidative damage [107]. Created in BioRender. https://BioRender.com/489727p (accessed on 16 September 2025).

## 5. Risk Factors and Oxidative Stress in HNSCC

The three major risk factors for HNSCC are tobacco smoking, alcohol consumption, and high-risk human papillomavirus (HR-HPV) infection [108]. These factors often act synergistically, markedly increasing HNSCC risk, as shown in a meta-analysis of 17 studies involving over 27,000 patients [109]. Although each factor operates through different mechanisms, all three disrupt the balance between ROS and antioxidant defenses, leading to oxidative stress, cellular damage, and ultimately carcinogenesis [41].

Alcohol metabolism contributes to ROS production through the conversion of ethanol to the carcinogenic metabolite acetaldehyde by enzymes such as cytochrome P450 2E1 (CYP2E1), alcohol dehydrogenase (ADH), and catalase. Induction of CYP2E1 not only increases acetaldehyde levels but also generates ROS, which can damage DNA, proteins, and lipids, thereby promoting tumorigenesis [110].

Tobacco smoke contains both a gas phase (<0.1 μm) and a tar (particulate) phase (0.1–1 μm), both of which are rich in free radicals. These radicals induce oxidative stress, leading to cellular damage and promoting cancer development [111].

In HPV-negative HNSCC, alcohol- and tobacco-induced ROS trigger dimerization of TM4SF19, stabilising GABPβ1 and activating YAP1 expression. Elevated YAP activity correlates with poor prognosis and therapy resistance [112].

HPV infection and oxidative stress have a bidirectional relationship. In the early stages of HPV infection, oxidative stress alters the local cellular environment in a way that facilitates viral integration by enhancing DNA damage and weakening DNA repair mechanisms [61]. Additionally, high levels of oxidative stress markers may reduce the clearance of HPV infection.

HPV also contributes to increased ROS production, primarily through the induction of chronic inflammation. Compared to HPV-negative HNSCC, HPV-positive HNSCC cells show higher activity in genes related to β-oxidation. This metabolic pathway produces hydrogen peroxide (H_2_O_2_), a reactive oxygen species, which can subsequently give rise to superoxide radicals (O_2_^• −^). These radicals may diffuse into the cytosol and generate highly reactive hydroxyl radicals (•OH) through Fenton and Haber–Weiss reactions [113].

The resulting hydroxyl radicals cause oxidative damage, as reflected by increased levels of malondialdehyde (MDA) and 4-hydroxynonenal (4-HNE), both key biomarkers of lipid peroxidation [114]. Notably, elevated MDA levels have been correlated with higher tumour burden and poorer clinical prognosis in HNSCC patients [115].

Additionally, HPV oncoproteins E6 and E7 contribute to ROS production through several mechanisms. These include activation of NOX2, an enzyme that generates ROS and leads to oxidative DNA damage [116]; inactivation of key antioxidant enzymes such as SOD2 and GPx, which reduces the cell’s capacity to neutralise ROS [117]; and promotion of the degradation of tumour suppressors p53 and retinoblastoma protein (pRb), weakening genomic protection and increasing genomic instability [118]. As a result, genomic instability and oxidative damage are more pronounced in HPV-positive HNSCC [41].

In summary, alcohol, tobacco, and high-risk HPV contribute to HNSCC development largely through the induction of oxidative stress. Understanding these mechanisms highlights the central role of redox homeostasis in tumour initiation and progression and points to potential opportunities for prevention and targeted therapeutic intervention.

## 6. ROS-Targeted Therapies and Translational Relevance

Targeting ROS is emerging as a novel approach for treating HNSCC, but it comes with both opportunities and challenges. The general idea is to disturb the fragile redox balance that cancer cells rely on for survival. Depending on the context, this can be done by pushing ROS levels high enough to induce cell death, or by lowering them to limit damage and slow tumour progression.

**Figure 3:**
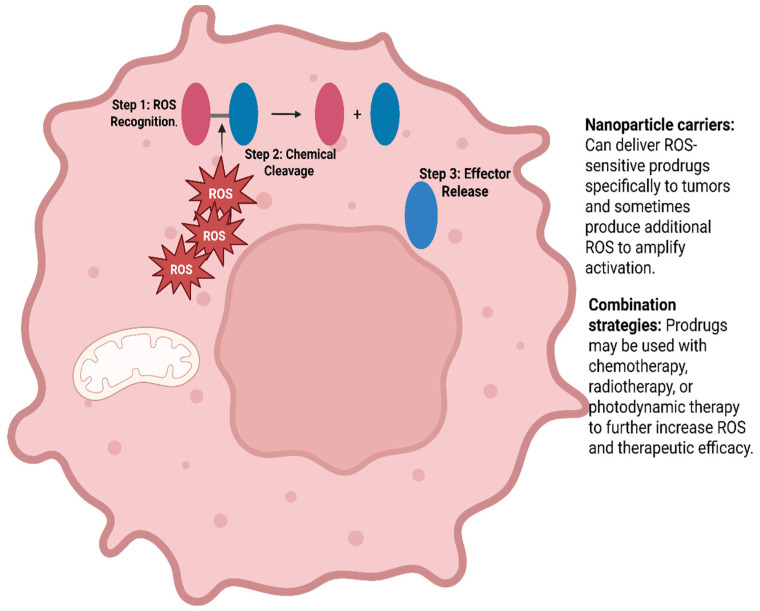
Mechanism of ROS-Activated Prodrugs Targeting Cancer Cells.

One way to exploit ROS is through ROS-inducing therapies. Many standard treatments, such as chemotherapy and radiotherapy, already work in part by increasing ROS and overwhelming the cancer cell’s defences [119]. More specific strategies include ROS-activated prodrugs, which are designed to stay inactive until they encounter high ROS concentrations like hydrogen peroxide. Once triggered, these drugs release a toxic component only inside the tumour, which helps limit damage to normal tissue [119,120] Figure 3.

Another promising area is the use of nanomaterials. Nanoparticles can be engineered to either generate ROS directly—such as in photodynamic (PDT), sonodynamic (SDT), or chemodynamic (CDT) therapy—or to carry ROS donors into tumour cells [120,121]. Some nanoparticle systems also work by depleting antioxidants or blocking antioxidant enzymes, which makes cancer cells less able to buffer oxidative stress [121].

From a clinical standpoint, the appeal of these approaches lies in their potential to sensitise tumours to conventional therapies. By disrupting redox balance, ROS-targeted strategies may help overcome resistance to chemotherapy and radiotherapy, while also reducing the dose-related toxicity of standard treatments [122,123].

Still, moving these strategies into the clinic is not straightforward. ROS biology is highly context-dependent [123], and delivering drugs selectively to tumours without affecting healthy tissues remains a major obstacle [120]. The tumour microenvironment adds another layer of complexity: hypoxia and high levels of glutathione (GSH), for example, can buffer ROS and make these therapies less effective [121].

Taken together, ROS-targeted therapies hold real promise for HNSCC, but their success will depend on overcoming the biological and technical barriers that currently limit translation.

Figure 3: ROS-activated prodrugs are engineered to exploit the elevated ROS levels in cancer cells. The drug remains chemically inert under normal physiological conditions, preventing toxicity in healthy tissues. The prodrug consists of a trigger (ROS-sensitive moiety), a cytotoxic effector, and a linker connecting the two. Step 1: ROS Recognition—The ROS-sensitive trigger reacts with elevated ROS in the tumour microenvironment. Step 2: Chemical Cleavage—ROS oxidizes the trigger, breaking the linker. Step 3: Effector Release—The cytotoxic drug is released in its active form inside the cancer cell. Created in BioRender. <Bani-Ahmad>, <Enas>. (2025) https://BioRender.com/itlh4cq (accessed on 28 September 2025).

## 7. Future Directions and Conclusions

Oxidative stress plays a multifaceted role in HNSCC, influencing tumour initiation, progression, and responses to therapy. ROS function as a double-edged sword: at moderate levels, they promote neoplastic transformation, proliferation, and survival, whereas at high levels, they trigger apoptosis and cell death. Cancer cells develop adaptive mechanisms to maintain redox balance, including the activation of transcriptional pathways and upregulation of antioxidant enzymes. These adaptations support tumour growth and contribute to resistance against chemotherapy and radiotherapy by neutralising drugs, modulating autophagy, and preventing apoptosis.

Understanding the interplay between ROS production, antioxidant defences, and the signalling networks they influence is essential for identifying new therapeutic targets. Strategies that disrupt tumour oxidative stress adaptations—either by increasing ROS to cytotoxic levels or inhibiting antioxidant defences—hold promise for sensitising HNSCC cells to treatment. Future research should focus on defining the context-dependent effects of ROS in HNSCC, clarifying the prognostic value of biomarkers such as GSH/GSSG ratios, and developing targeted interventions that exploit vulnerabilities created by altered redox homeostasis.

## Figures and Tables

**Table 2:** Antioxidant Defence Mechanisms in Normal vs. HNSCC Tissues.

Antioxidant	Normal Function	Status in HNSCC	Clinical Relevance in HNSCC	Type	Ref
GSH	Is the major thiol-based defense system against oxidative and electrophilic stress markers in the cell	Reduced levels in blood and elevated levels in tumour tissues observed	Potential biomarker for tumour behaviours and patient outcomes	Nonenzymatic system	[72,73]
*β*-carotenes	Present a robust antioxidant capacity that contributes to protecting the body against the effects of ROS.	Often lower than in healthy individuals	A diet rich in carotenoids, including β-carotene, may be associated with a reduced risk of developing HNSCC.	Nonenzymatic system	[74,75,76]
Vitamin E (*α-*, *β*-, *γ*-, and *δ*-tocopherols)	Scavenges peroxyl radicals by donating hydrogen from the phenolic group on the chromanol ring and terminates the oxidation of polyunsaturated fatty acids.	Vitamin E levels may be reduced, and this deficiency may play a role in the development of second primary tumours.	Inverse association between vitamin E intake and HNSCC risk, results are inconsistent and may vary depending on the source of vitamin E and the specific type of cancer.	Nonenzymatic system	[77,78,79]
Vitamin C (ascorbic acid)	It is a potent antioxidant capable of preventing oxidative damage and lipid peroxidation induced by peroxide radicals, can reduce unstable biomolecules (nitrogen, oxygen, and sulfur radicals), and has the function of regenerating Vitamin E and other antioxidants in the organism	Vitamin C status generally decreases.	Have a protective effect against HNSCC.	Nonenzymatic system	[80,81,82]
Uric acid	End product of purine metabolism via xanthine oxidase, has a dual role in redox biology. It acts as an antioxidant by scavenging ROS and preventing lipid peroxidation. However, at high levels, uric acid may become harmful, contributing to induced oxidative stress.	No significant differences in salivary uric acid levels between HNSCC patients and healthy controls	Uric acid levels, both pre- and post-treatment, can be associated with outcomes like metastasis and overall survival.	Nonenzymatic system	[83,84]
SOD	All forms of SOD rapidly dismutase superoxide to the more stable ROS (H_2_O_2_), which is then converted to water and oxygen	Levels of superoxide dismutase (SOD) can increase	Increased SOD2 expression has been linked to larger tumour size, nodal involvement, and earlier relapse.	The enzymatic system	[85,86]
Trx system comprises NADPH, thioredoxin reductase (TrxR), and Trx	The Trx system provides the electrons to thiol-dependent peroxidases Removes ROS with a fast reaction rate.	Increased expression and activity	Associated with tumour progression, resistance to therapy, and immune escape.	The enzymatic system	[87,88]
NQO1	NQO1 can reduce ubiquinone and vitamin E quinone to their antioxidant forms and reduce superoxide directly, suggesting a primary protective role	Typically overexpressed	Overexpression of NQO1 in HNSCC is linked to increased cancer cell proliferation, migration, and resistance to certain therapies.	The enzymatic system	[89,90]
HO-1	Can prevent excessive oxidation of lipids and proteins by scavenging hydroxyl-free radicals, singlet oxygen, and superoxide anions and play an effective role in anti-inflammation, antioxidation, and antiapoptotic	Exhibits a complex and context-dependent role. Some studies have shown that HO-1 is expressed in the majority of HNSCCs and that its expression is higher in tumours compared to normal or non-malignant adjacent tissue.	While HO-1 can promote cancer progression and resistance to therapy in some contexts, it also plays a role in maintaining cellular homeostasis and potentially inhibiting tumour growth in others	The enzymatic system	[91,92,93,94,95]
Nrf-2	Main regulator of the antioxidant system	Generally increased	Correlate with more aggressive tumour behaviour and poorer patient outcomes.	The antioxidant transcription factors	[96,97]
FOXO	Regulation of oxidative stress, attenuating ROS	The role of FOXO1 in HNSCC is complex and can vary.	FOXO1 acts as a tumour promoter or suppressor in HNSCC. While some studies suggest it can promote tumour progression through mechanisms like increased M2 macrophage infiltration, other studies indicate it can act as a tumour suppressor, particularly when downregulated or when its downstream pathways are activated.	The antioxidant transcription factors	[98]

Heme Oxygenase-1: HO-1, Nuclear factor 2 related to erythroid factor 2: Nrf-2, Forkhead box O: FOXO.

## Data Availability

No new data were created or analyzed in this study. Data sharing is not applicable to this article.
